# Plasma-Enabled Pd/C Catalysts with Rich Carbon Defects for High-Performance Phenol Selective Hydrogenation

**DOI:** 10.3390/nano16010048

**Published:** 2025-12-29

**Authors:** Yu Zhang, Ying Xin, Lizheng Tang, Shihao Cui, Hongling Duan, Qingshan Zhao

**Affiliations:** State Key Laboratory of Heavy Oil Processing, College of Chemistry and Chemical Engineering, China University of Petroleum (East China), Qingdao 266580, China; s24030086@s.upc.edu.cn (Y.Z.); z24030047@s.upc.edu.cn (Y.X.); z25030066@s.upc.edu.cn (L.T.); cuishihao001124@163.com (S.C.)

**Keywords:** Pd/C catalyst, plasma treatment, selective hydrogenation, carbon defects

## Abstract

The selective hydrogenation of phenol to cyclohexanone is a pivotal reaction for producing nylon precursors. Conventional Pd/C catalysts, however, suffer from weak metal–support interactions, leading to size heterogeneity and agglomeration of Pd nanoparticles, which degrades their activity and stability. Herein, we report a facile argon plasma treatment to engineer rich defects on an activated carbon (AC) support, resulting in a highly dispersed and stable catalyst (denoted as PL-Pd@AC_Ar_). Characterization results indicate that the abundant carbon defects in PL-Pd@AC_Ar_ enhance the anchoring of Pd precursors, ensure the uniform dispersion of Pd nanoparticles, and effectively modulate their electronic structure. Consequently, the plasma-enabled PL-Pd@AC_Ar_ catalyst achieves 99.9% phenol conversion with 97% selectivity to cyclohexanone at a mild temperature of 70 °C and maintains exceptional stability over six consecutive cycles. This work provides a robust and efficient strategy for the surface engineering of carbon supports to design high-performance hydrogenation catalysts.

## 1. Introduction

Cyclohexanone serves as a pivotal intermediate in the nylon industry, mainly utilized for the synthesis of caprolactam and adipic acid [1]. Current industrial production routes encompass cyclohexane oxidation, benzene hydrogenation, cyclohexanol oxidation, and phenol hydrogenation [2,3,4,5]. Among these approaches, the selective hydrogenation of phenol to cyclohexanone is regarded as highly consistent with green chemistry principles, as it eliminates the need for oxidants and achieves high atom economy. In this regard, Pd/C catalysts have garnered extensive attention due to the superior hydrogenation activity of Pd and the excellent stability of the carbon support [6,7,8,9,10,11]. However, the practical application of Pd/C catalysts is frequently hampered by the inert surface and limited diversity of functional groups of commercial activated carbon, which results in weak metal–support interaction (MSI). This weakness tends to induce the size heterogeneity, agglomeration, and even leaching of Pd nanoparticles during preparation or reaction processes, thereby reducing metal utilization efficiency, reducing the number of active sites, and impairing catalytic stability [12,13,14]. To address these challenges, researchers have focused on enhancing MSI through surface modification of the support. Strategies such as introducing oxygen-containing groups via nitric acid oxidation or incorporating heteroatoms like nitrogen have demonstrated potential in improving Pd dispersion and catalytic performance [15,16,17,18]. Nonetheless, these methods often exhibit drawbacks including the generation of acidic waste, prolonged processing durations, potential structural damage to the carbon framework, and inadequate control over the intrinsic defect structure. Therefore, the development of green and efficient modification strategies is crucial for advancing the next generation of high-performance Pd/C catalysts.

In general, plasma is primarily generated via energetic activation of gaseous substances, leading to either partial or complete ionisation of the gas into unbound free electrons, ions, radicals, and other excited species [19]. The energy input methods include ultraviolet radiation, electromagnetic field excitation, high-temperature heating, X-rays and other techniques [20]. Based on the system temperature, plasma can be categorized into high-temperature plasma and low-temperature plasma [21]. The gas in high-temperature plasma is almost fully ionized, with the electron temperature (Te), ion temperature (Ti), neutral particle temperature (Tn), and macroscopic gas temperature (Tg) being equal. It exists in a state of complete thermodynamic equilibrium, with a gas temperature exceeding 6000 °C, such as corona plasma and controlled thermonuclear fusion plasma [22]. In low-temperature plasma, the gas undergoes partial ionization, resulting in a significant discrepancy between T_e_, T_i_, T_n_, and T_g_ [23]. Furthermore, low-temperature plasma can be further subdivided into thermal plasma and non-thermal plasma according to thermodynamic equilibrium states. Thermal plasma is a local thermodynamic equilibrium plasma satisfying T_e_ ≈ T_i_ ≈ T_n_ > T_g_, while the non-thermal plasma is a non-thermodynamic equilibrium plasma characterized by T_e_ > T_i_ ≈ T_n_ ≈ T_g_.

Non-thermal plasma has been extensively applied in the surface treatment or modification of catalysts [23]. The objectives of such treatment include the generation of surface vacancies [24] and defects [25], metal doping [26], optimization of surface morphology [27], increment of active sites [28], and reconstruction of surface functional groups [29]. This technique allows for the modulation of surface morphology through momentum transfer from high-energy electrons and ions under near-ambient temperature conditions. Meanwhile, the abundant reactive radicals and excited species generated in the plasma can induce surface chemical reactions, thereby constructing defect structures that effectively enhance the interaction between the metal and the support, defined as the strong metal–support interaction (SMSI) effect [30,31]. In carbon materials, the introduced defect sites act as efficient anchoring points to firmly immobilize metal nanoparticles, thus improving the interfacial stability of the catalyst [32,33]. Furthermore, defect sites generally exhibit higher electron cloud density than regular carbon atoms, which not only facilitates the capture and stabilization of metal nanoparticles but also promotes electron transfer and substrate adsorption during catalytic reactions, collectively enhancing the overall catalytic performance [34,35]. For example, Chen et al. constructed Ni_12_P_5_-Ni_4_Nb_5_P_4_ heterointerfaces on carbon cloth using DBD plasma, greatly enhancing its HER activity and stability beyond the Pt/C benchmark [36]. Rao et al. developed a Co single-atom catalyst (Co-SAC/NC) with a high Co loading (2.5 wt%) via an efficient “plasma-bombing” strategy, in this approach, nitrogen plasma simultaneously etched defects into the nitrogen-doped carbon support and anchored isolated Co atoms onto these defect sites. This defect-rich structure enabled robust ORR activity in alkaline media and excellent performance in zinc–air batteries [37]. Owing to these merits, plasma treatment provides an efficient and environmentally benign strategy for the surface engineering of carbon-based supports, laying a solid technological foundation for achieving uniform loading of active metals and the rational design of high performance catalysts.

Leveraging the unique advantages of plasma technology in surface modification, we propose argon plasma treatment to fabricate a high-performance Pd/C catalyst (denoted as PL-Pd@AC_Ar_) by regulating the defect structure and chemical environment of the activated carbon (AC) support. Under remarkably milder reaction conditions compared to those reported in the literature, the plasma-engineered PL-Pd@AC_Ar_ catalyst achieves 99.9% phenol conversion with 97% selectivity in the hydrogenation reaction, exhibiting significantly enhanced catalytic activity relative to the untreated Pd@AC_0_ catalyst. The performance enhancement is primarily ascribed to the plasma-induced abundant carbon defects, which not only serve as robust and uniform anchoring sites for Pd nanoparticles but also enhance metal–support interaction for promoted catalytic characteristics. This work develops a green and efficient modification strategy for the surface engineering of carbon-based supports and offers valuable insights for the rational design of high-performance supported metal catalysts.

## 2. Materials and Methods

### 2.1. Materials

Unless otherwise stated, all chemicals shall be used in the state as they were received. Nitric acid (HNO₃, AR) and hydrochloric acid (HCl, AR) were purchased from Sinopharm Chemical Reagent Co., Ltd. (Qingdao, China). Palladium chloride (PdCl₂, 98.0%), cyclohexanone (AR), and sodium carbonate (Na₂CO₃, 99.8%) were obtained from Aladdin Chemical Reagent Co., Ltd. (Qingdao, China). Cyclohexanol (AR) was supplied by Aladdin Chemical Reagent Co., Ltd. (Jinan, China). Phenol (AR) was acquired from Aladdin Chemical Reagent Co., Ltd. (Shanghai, China). Activated carbon material was purchased from Arkema (Colombes, France).

### 2.2. Preparation of Catalysts

#### 2.2.1. Preparation of Argon Plasma-Treated Activated Carbon (AC-PL_Ar_)

Plasma treatments were conducted using a SAT-5D plasma cleaning system, which operates based on the principle of capacitively coupled plasma (CCP). Untreated AC was uniformly spread onto a watch glass, which was then placed in a radio frequency (RF) plasma chamber. The chamber was evacuated to a vacuum pressure of 60 Pa and held at this pressure for 5 min. Subsequently, high-purity argon gas was introduced into the chamber, and a plasma was ignited at an RF power of 200 W to perform surface modification on the activated carbon. After the plasma treatment, the activated carbon was repeatedly washed with ultrapure water three times to remove potential residual impurities and surface reaction by-products, ultimately yielding argon plasma-modified activated carbon (denoted as PL-AC_Ar_). For comparison, under identical instrument parameters, the reaction atmosphere was switched to high-purity oxygen gas to prepare an oxygen plasma-treated activated carbon sample (denoted as PL-AC_O2_).

#### 2.2.2. Preparation of PL-Pd@AC_Ar_ Catalyst

The PL-Pd@AC_Ar_ catalyst was synthesized via a deposition-precipitation method, wherein the PL-AC_Ar_ support was first ultrasonically dispersed in deionized water to form a homogeneous suspension while PdCl_2_ corresponding to 5 wt% Pd loading was dissolved in an HCl solution under ultrasonication, followed by added dropwise into the suspension and subsequent neutralization of the mixture pH through controlled addition of Na_2_CO_3_ solution. The final slurry was continuously stirred at room temperature for 24 h to ensure the complete precipitation of Pd(OH)_2_. Subsequently, the resulting precipitate (denoted as Pd^2+^@AC) was subjected to H_2_ reduction, whereby Pd^2+^ ions were progressively reduced to metallic Pd^0^ atoms, followed by washing and drying to obtain the target catalyst. To systematically investigate the support modification effects, reference catalysts Pd@AC_0_ and PL-Pd@AC_O2_ were prepared following identical procedures employing pristine activated carbon (AC_0_) and oxygen plasma-modified activated carbon (PL-AC_O2_) as supports, respectively.

### 2.3. Catalytic Performance of Phenol Hydrogenation

The catalyst activity evaluation was conducted in a micro high-pressure reaction apparatus. First, 0.5 mmol of phenol, 5 mg of catalyst, and 10 mL of cyclohexane were sequentially added to the liner of the reaction kettle. To ensure an oxygen-free environment, the autoclave was pressurized with H_2_ three times to effectively remove the existing air. The hydrogen pressure was then adjusted to the preset value, and the reaction system was heated to the target temperature. The reaction was carried out under constant stirring conditions. After the hydrogenation reaction was completed, the product was sampled for analysis. The product was analyzed using a BF-2002 gas chromatograph.

The mass fractions of phenol and cyclohexanone in the product could be calculated according to the calibration curve equation of the external standard method. The formulas for calculating the phenol conversion (C) and cyclohexanone selectivity (S) in the hydrogenation reaction are given by Equations (1) and (2):

(1) $$phenol\;conversion=\frac{Mole\;of\;phenol\;reacted}{Mole\;of\;initial\;phenol}$$

(2) $$cyclohexanone\;selectivity=\frac{Mole\;of\;cyclohexanone}{Mole\;of\;phenol\;converted}$$

### 2.4. Catalyst Characterization

X-ray diffraction (XRD) analysis was performed to characterize the crystal structures of the catalysts using an X’PertPro MPD polycrystalline powder X-ray diffractometer (Malvern Panalytical, Almelo, The Netherlands). Electron paramagnetic resonance (EPR) measurements were conducted on an A300 spectrometer (Bruker Corporation, Billerica, MA, USA) in the X-band (~9.5 GHz), with a 100 kHz magnetic field modulation and phase-locked amplification technique employed to enhance detection sensitivity. Raman spectroscopy was carried out using an inVia Reflex 200 confocal micro-Raman spectrometer (Renishaw plc, Wotton-under-Edge, UK) for structural characterization of the materials. Scanning electron microscopy (SEM) images were obtained with a Sigma300 field-emission scanning electron microscope (Carl Zeiss AG, Oberkochen, Germany). Transmission electron microscopy (TEM) images were acquired using a Tecnai F20 high-resolution transmission electron microscope (FEI Company, Hillsboro, OR, USA). X-ray photoelectron spectroscopy (XPS) was carried out using an ESCALAB 250Xi X-ray photoelectron spectrometer (Thermo Fisher Scientific, Waltham, MA, USA) to analyze the surface chemical properties of the materials. 

## 3. Results and Discussion

**Figure 1:**
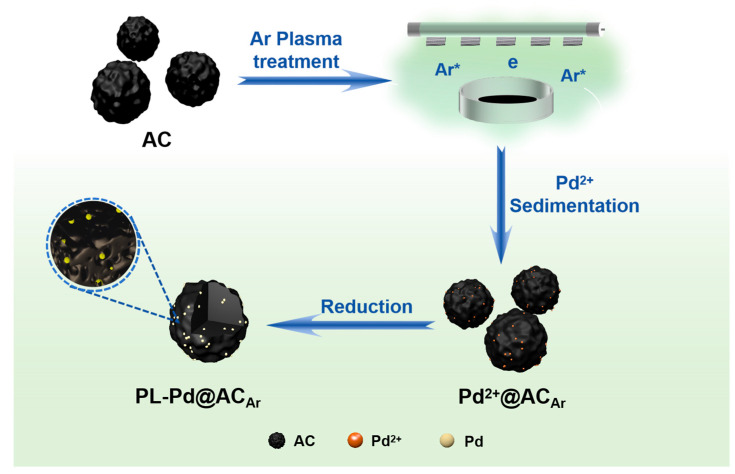
Schematic illustration of the preparation procedure of the PL-Pd@AC_Ar_ catalyst.

**Figure 2:**
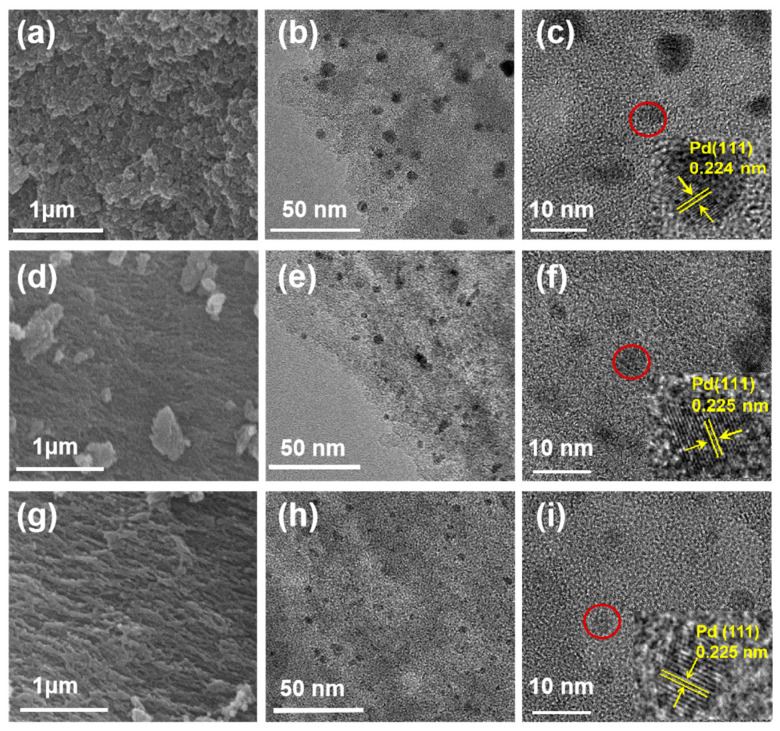
(**a**) SEM image of AC_0_ (scale bar is 1 μm); (**b**) TEM images of Pd@AC_0_ (scale bar is 50 nm); (**c**) TEM images of Pd@AC_0_ (scale bar is 10 nm); (**d**) SEM image of PL-AC_O2_ (scale bar is 1 μm); (**e**) TEM images of PL-Pd@AC_O2_ (scale bar is 50 nm); (**f**) TEM images of PL-Pd@AC_O2_ (scale bar is 10 nm); (**g**) SEM image of PL-AC_Ar_ (scale bar is 1 μm); (**h**) TEM images of PL-Pd@AC_Ar_ (scale bar is 50 nm); (**i**) TEM images of PL-Pd@AC_Ar_ (scale bar is 10 nm).

Figure 1 illustrates the argon plasma treatment strategy for support modification and the fabrication process of the PL-Pd@AC_Ar_ catalyst. The pristine AC was subjected to radio-frequency argon plasma treatment. During this process, high-energy argon ions bombard the carbon surface, transferring momentum to surface atoms and leading to physical sputtering, which can generates lattice vacancy defects [38]. To investigate the effects of different plasma treatments on the support structure and metal dispersion, the catalysts were characterized by SEM and TEM. SEM results indicate that different gas atmospheres exert a significant influence on the surface morphology of the supports. Specifically, Figure 2a shows the untreated AC_0_ sample, which exhibits a disordered surface structure and uneven pore distribution. When comparing the oxygen plasma-treated PL-AC_O2_ (Figure 2d) with the argon plasma-treated PL-AC_Ar_ (Figure 2g), the latter displays a more uniform pore structure. The distribution of Pd nanoparticles were further observed via TEM (Figure 2 and Figure S1). Figure 2b,c correspond to the Pd@AC_0_ catalyst, Figure 2e,f to PL-Pd@AC_O2_, and Figure 2h,i to PL-Pd@AC_Ar_. TEM images reveal that Pd nanoparticles in all catalysts exhibit a spherical morphology, with a lattice fringe spacing of 0.23 nm, that is consistent with the characteristic (111) crystal plane of Pd. Particle size analysis results (Figure S2) demonstrate that the PL-Pd@AC_Ar_ catalyst has the smallest average Pd particle size (3.49 nm) with the most uniform distribution. In contrast, PL-Pd@AC_O2_ and Pd@AC_0_ have larger average Pd particle sizes of 4.09 nm and 6.05 nm, respectively, and exhibit obvious agglomeration, as shown in Figure S1.

**Figure 3:**
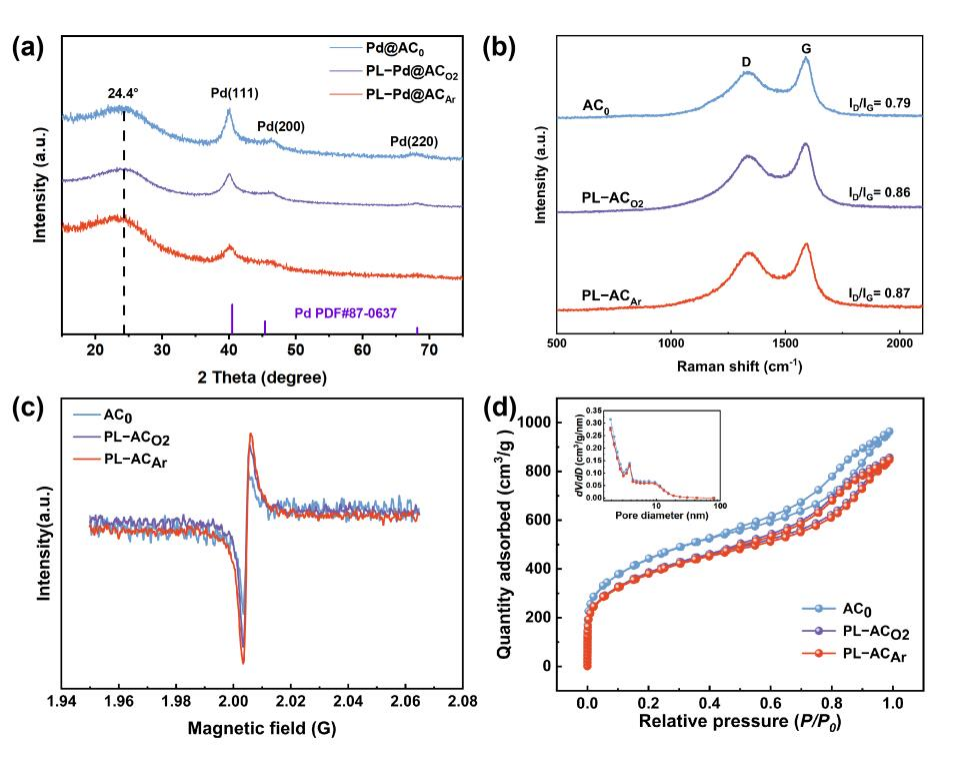
(**a**) XRD patterns of Pd@AC_0_, PL-Pd@AC_O2_, and PL-Pd@AC_Ar_; (**b**) Raman spectra of AC_0_, PL-AC_O2_, and PL-AC_Ar_; (**c**) EPR spectra of AC_0_, PL-AC_O2_, and PL-AC_Ar_; (**d**) N_2_ adsorption–desorption isotherms and pore size distribution curves of AC_0_, PL-AC_O2_, and PL-AC_Ar_.

The crystalline structures of the catalysts were characterized by XRD, as presented in Figure 3a. The XRD patterns exhibit distinct diffraction peaks at 40.2°, 46.5°, and 68.3°, corresponding to the (111), (200), and (220) planes of metallic Pd, respectively, confirming the successful loading of crystalline Pd on all supports [39]. Further analysis of the XRD patterns reveals differences in the Pd crystallite characteristics among the samples. The Pd@AC_0_ sample shows the sharpest and most intense Pd (111) diffraction peak at 40.2° with the smallest full width at half maximum (FWHM). According to the Scherrer equation [40], this indicates the presence of larger Pd crystallites in Pd@AC_0_. In contrast, the Pd (111) peaks of the plasma-pretreated samples, PL-Pd@AC_O2_ and PL-Pd@AC_Ar_, are significantly broadened and with reduced intensity, which corresponds to smaller Pd crystallite sizes as calculated via the Scherrer equation. The trend in Pd crystallite size derived from XRD is consistent with the TEM observations (Figure 2). The structure of the carbon support was further analyzed by Raman spectroscopy. As shown in Figure 3b, the I_D_/I_G_ ratios of the three AC samples are 0.79 (AC_0_), 0.86 (PL-AC_O2_), and 0.87 (PL-AC_Ar_), respectively. Notably, the highest I_D_/I_G_ ratio observed for the argon plasma-treated PL-AC_Ar_ sample indicates that argon plasma treatment most effectively introduces defect sites on the carbon surface [41].

To further investigate the effects of plasma treatment under different atmospheres on the defect sites and specific surface areas of the supports, EPR and BET characterizations were performed on the prepared supports (Figure 3c,d). As shown in Figure 3c, the EPR spectra of all three AC samples exhibit a Lorentzian line shape with a g-factor of 2.003, which is characteristic of unpaired electrons on carbon atoms. The peak intensity is significantly enhanced in the argon plasma treatment sample (PL-AC_Ar_), which shows the highest concentration of defect sites, consistent with the Raman characterization results. Furthermore, Figure 3d presents the N_2_ adsorption–desorption isotherms of three AC samples, with the inset showing the corresponding pore size distribution curves. All three samples exhibit type IV isotherms with distinct hysteresis loops, demonstrating the coexistence of micropores and mesopores. Plasma treatment did not significantly alter the pore size distribution. However, the specific surface area decreased after plasma treatment, with values of 1563 m^2^/g for AC_0_, 1367 m^2^/g for PL-AC_O2_, and 1347 m^2^/g for PL-AC_Ar_. This reduction can be attributed to the high-energy physical bombardment effect of plasma treatment. On one hand, the process effectively introduces carbon vacancies and defects at the atomic scale, which is conducive to the formation of more active sites. On the other hand, it may also involve surface etching and redeposition of carbon species, leading to partial blockage or coverage of micropore entrances, resulting in a decrease in specific surface area. Overall, although argon plasma treatment slightly reduces the macroscopic specific surface area of the support, it constructs a high concentration of surface defects, significantly enhancing the anchoring capability of the support toward Pd nanoparticles.

**Figure 4:**
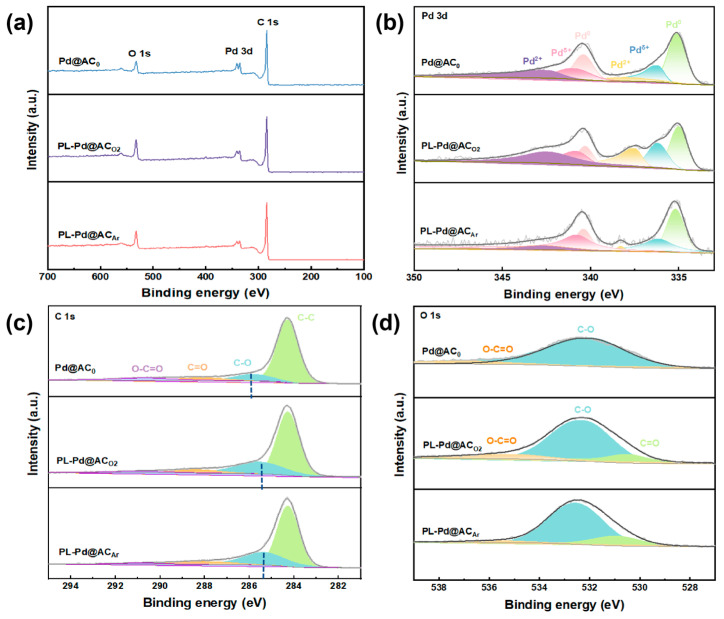
(**a**) XPS survey spectra, (**b**) Pd 3d spectra, (**c**) C 1s survey spectra, (**d**) O 1s spectra of Pd@AC_0_, PL-Pd@AC_O2_, and PL-Pd@AC_Ar_ catalysts.

XPS was further performed on the Pd@AC_0_, PL-Pd@AC_O2_, and PL-Pd@AC_Ar_ catalysts. The survey spectra (Figure 4a) confirm the presence of C, O and Pd elements in all three samples. As shown in the high-resolution Pd 3d spectra (Figure 4b), characteristic peaks corresponding to Pd^2+^, Pd^δ+^, and Pd^0^ are observed, with the PL-Pd@AC_Ar_ catalyst exhibiting significantly higher contents of Pd^δ+^ and Pd^0^. The C 1s spectra (Figure 4c) show that in the plasma-treated sample, the C-H bond peak shifts toward lower binding energy by 0.1–0.2 eV toward lower binding energy, accompanied by a decrease in the intensity of the C-C bond peak, consistent with the formation of defect structures within the carbon framework as confirmed by EPR analysis. These defects provide abundant anchoring sites. ICP analysis further confirms this, showing higher Pd loadings on PL-Pd@AC_Ar_ (4.91 wt%) and PL-Pd@AC_O2_ (4.86 wt%) compared to that on Pd@AC_0_ (3.40 wt%). Furthermore, the O 1s spectra (Figure 4d) reveal that oxygen exists primarily in the forms of Pd-O, O-C, and -OH. The oxygen content increases from 18.9% in the untreated sample to 28.9% and 27.7% after treatment, which is attributed to reactions between adsorbed water/oxygen and plasma-generated active species. The introduced oxygen species can not only enhance the hydrophilicity of the catalyst but also modulate the adsorption/desorption behavior of reactants and products. Meanwhile, the increased surface oxygen content elevates the surface energy of the AC support, particularly around the defect sites, thereby strengthening the anchoring interaction between the support and Pd nanoparticles. Integrating the XPS, EPR, and ICP results, it can be concluded that the carbon vacancies generated by plasma treatment facilitate the uniform loading of Pd and contribute to the modulation of the electronic structure of Pd.

**Figure 5:**
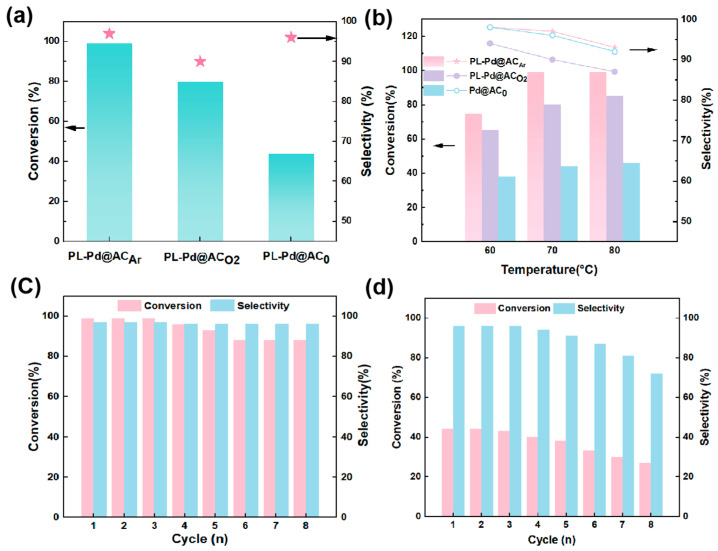
(**a**) The catalytic performance of the three catalysts at 70 °C; (**b**) Catalytic performance of the three catalysts in phenol hydrogenation as a function of temperature; (**c**) Reusability of PL-Pd@AC_Ar_; (**d**) Reusability of PL-Pd@AC_0_.

The effect of plasma treatments on the catalytic hydrogenation performance of the catalysts was investigated. As presented in Figure 5a and Figure S3, the Pd@AC_0_ catalyst without plasma treatment exhibited a conversion of only 46% in the hydrogenation of phenol to cyclohexanone, while the plasma treated catalysts showed remarkably promoted catalytic activity, the PL-Pd@AC_O2_ catalyst pretreated with oxygen plasma achieved a conversion of 80%, and the PL-Pd@AC_Ar_ catalyst pretreated with argon plasma showed a significantly promoted catalytic performance, with the phenol conversion increased to 99%. The catalytic performance of the catalyst under different temperature conditions was further explored. Under the reaction conditions of 70 °C and 1 MPa H_2_, the PL-Pd@AC_Ar_ catalyst achieved a phenol conversion of 99.9% and a cyclohexanone selectivity as high as 97.0% (Figure 5b). The high activity and selectivity of this catalyst at relatively low temperature are attributed to the increased carbon defect concentration, optimized pore structure, and improved dispersion and electronic modulation of Pd nanoparticles induced by argon plasma treatment. Moreover, as summarized in Table S1, PL-Pd@AC_Ar_ also demonstrated excellent hydrogenation activity toward various hydroxy-aromatic compounds, demonstrating outstanding substrate applicability and potential industrial application prospects.

In reusability tests, PL-Pd@AC_Ar_ and PL-Pd@AC_0_ catalysts exhibited distinctly different stability behaviors, highlighting the significant advantages of plasma treatment in structural engineering and catalytic durability. PL-Pd@AC_Ar_ demonstrated exceptional cycling stability, maintaining high cyclohexanone selectivity throughout all cycles. Although a slight decline in phenol conversion was observed over the first eight cycles, the catalytic performance stabilized after the sixth cycle. Post-reuse XPS characterization (Figure S4) reveals the disappearance of the Pd^δ+^ species and an increase in Pd^2+^ content from 21% to 45%, a valence state transition crucial for sustaining catalytic activity. In contrast, PL-Pd@AC_0_ exhibited lower initial catalytic activity and inferior cycling stability, with conversion continuously declining over repeated cycles. This rapid deactivation is primarily attributed to severe agglomeration or migration of active species during the reaction process, leading to irreversible loss of active sites. In summary, the structural robustness and sustained performance of PL-Pd@AC_Ar_ underscore its great potential for industrial application, whereas the rapid deactivation of Pd@AC_0_ further emphasizes the importance of rational support engineering in the development of highly stable supported metal catalysts.

The PL-Pd@ACAr catalyst demonstrates excellent catalytic performance in the selective hydrogenation of phenol to cyclohexanone, demonstrating remarkable advantages over other reported Pd-based systems summarized in Table 1 [42,43,44,45,46,47,48,49,50,51,52]. Under mild reaction conditions (70 °C, 1 MPa H_2_, 2 h) with an extremely low Pd loading, the catalyst achieves 99.9% phenol conversion and 97% cyclohexanone selectivity. Compared with the non-plasma-treated reference catalyst Pd@AC_0_ (Entry 3), PL-Pd@AC_Ar_ shows significantly enhanced catalytic activity, increasing the conversion from 44.5% to 99.9% while retaining high cyclohexanone selectivity. Furthermore, PL-Pd@AC_Ar_ also presents notable advantages relative to other reported Pd-based catalytic systems. For instance, although the MOF-derived, N-doped catalyst Pd@CN-H (Entry 7) reaches 99.8% conversion at 80 °C, its selectivity is only 90.9%, and its synthesis depends on pre-formed MOF precursors. The two-dimensional carbon-supported Pd@CN(1:3)-650 (Entry 13), despite possessing a high specific surface area and abundant defect structures, requires complex preparation procedures and a substantially higher Pd loading of 0.16 wt%. The fibrous catalyst Pd@CN/SiNFs (Entry 4) enables facile separation but demands a higher Pd loading and a reaction temperature of 100 °C to sustain its activity. Lastly, the supported composite oxide Pd@ZrO_2_/AC (Entry 5) achieves complete phenol conversion but with a selectivity of only 88.3%. In summary, PL-Pd@AC_Ar_ stands out as an advanced catalytic system among reported catalysts for selective phenol hydrogenation. The plasma treatment adopted herein avoids high-temperature calcination or complex heteroatom doping processes, thereby providing a new avenue for the design of efficient hydrogenation catalysts with low noble-metal loadings.

## 4. Conclusions

In conclusion, a highly dispersed and stable PL-Pd@AC_Ar_ catalyst was successfully fabricated via a facile argon plasma treatment strategy for the enhanced selective hydrogenation of phenol to cyclohexanone. Argon plasma treatment effectively introduced carbon defects and optimized the pore structure without damaging the carbon framework. These structural modifications not only facilitate the uniform dispersion of Pd nanoparticles but also modulate their electronic structure. As a result, the PL-Pd@AC_Ar_ catalyst achieves nearly complete phenol conversion (99.9%) with high cyclohexanone selectivity (97%) under mild reaction conditions (70 °C, 1 MPa H_2_). Moreover, it maintains excellent catalytic stability over six consecutive reaction cycles, demonstrating promising potential for industrial application. This study validates plasma-induced surface engineering as a simple yet effective strategy for the preparation of high-performance carbon-supported metal catalysts, providing both a practical design for phenol hydrogenation and a general approach for the development of advanced catalytic systems.

## Figures and Tables

**Table 1:** Comparison of the Catalytic Performance of Phenol Hydrogenation over Pd-Based Catalysts.

Entry	Catalyst	Reaction Conditions [n_Pd_:n_Phenol_ (%), Temperature (°C), Time (h), Pressure (MPa)]	Conversion	Selectivity	Ref.
1	PL-Pd@AC_Ar_	0.0047, 70 °C, 2 h, 1 MPa	99.9	97	this work
2	PL-Pd@AC_O2_	0.0047, 70 °C, 2 h, 1 MPa	94	91	this work
3	Pd@AC_0_	0.0047, 70 °C, 2 h, 1 MPa	44.5	96.3	this work
4	Pd@CN/SiNFs	0.0090, 100 °C, 1 h, 0.1 MPa	97.5	96.1	[42]
5	Pd/@-ZrO_2_/AC (500)	0.0070, 80 °C, 3 h, 0.7 MPa	100	88.3	[43]
6	Pd/AC-600	0.0221, 80 °C, 1 h, 0.1 MPa	88.3	96	[44]
7	Pd@CN-H	0.0090, 80 °C, 0.83 h, 0.1 MPa	99.8	90.9	[45]
8	Pd@CN	0.0341, 80 °C, 1 h, 0.1 MPa	68.4	97.6	[46]
9	Pd@ZCNFs-20	0.0284, 80 °C, 0.5 h, 0.1 MPa	78.8	95	[47]
10	Pd/N4.8-meso-CNRs	0.0330, 40 °C, 3 h, 0.1 MPa	93.2	97.3	[48]
11	Pd/C-W	0.0487, 80 °C, 0.33 h, 0.31 MPa	97.2	97.3	[49]
12	Pd/SiO_2_-2	0.0033, 120 °C, 1.5 h, 0.3 MPa	80.6	92.1	[50]
13	Pd@CN(1:3)- 650	0.1600, 80 °C, 2 h, 0.1 MPa	94.0	94.7	[51]
14	Pd/Co_3_O_4_-H	0.0040, 80 °C, 4.5 h, 0.04 MPa	96	0	[52]

## Data Availability

Data are contained within the article.

## Supplementary Materials

The following supporting information can be downloaded at: https://www.mdpi.com/article/10.3390/nano16010048/s1, Figure S1: (a) TEM images of Pd@AC_0_; (b) TEM images of PL-Pd@AC_O2_; (c) TEM images of PL-Pd@AC_Ar_; Figure S2: Particle size distribution histograms showing the average sizes of (a) Pd@AC_0_, (b) PL-Pd@AC_O2_, (c) PL-Pd@AC_Ar_ Figure S3: Effect of different plasma pretreatments on catalytic performance as a function of time: (a) Air plasma, (b) O_2_ plasma, (c) Ar plasma. Figure S4: (a) XPS survey scan and (b) Pd 3d high-resolution spectrum of the recycled PL-Pd@AC_Ar_ catalyst. Table S1: Hydrogenation performance of PL-Pd@AC_Ar_ for various hydroxyaromatic compounds.
