# Supplementary material for: Plasma-Enabled Pd/C Catalysts with Rich Carbon Defects for High-Performance Phenol Selective Hydrogenation
Source: Nanomaterials (Basel). 2025 Dec 29;16(1):48. doi: 10.3390/nano16010048 (PMC12787857; doi:10.3390/nano16010048)
Supplement: Supplementary file 1 [file nanomaterials-16-00048-s001.zip › nanomaterials-4027422-supplementary.pdf]

nanomaterials

Supplementary Information

Plasma Treatment Enabling High-Performance Pd/C Catalysts with Carbon Defects in Phenol Selective Hydrogenation

Yu Zhang, Ying Xin, Lizheng Tang, Shihao Cui, Hongling Duan*, and Qingshan Zhao*

State Key Laboratory of Heavy Oil Processing, College of Chemistry and Chemical Engineering, China University of Petroleum (East China), Qingdao 266580, China; s24030086@s.upc.edu.cn (Y.Z.); z24030047@s.upc.edu.cn (Y.X.); z25030066@s.upc.edu.cn (L.T.); cuishihao001124@163.com (S. C.)

* Correspondence: qszhao@upc.edu.cn; dr_hs00@163.com

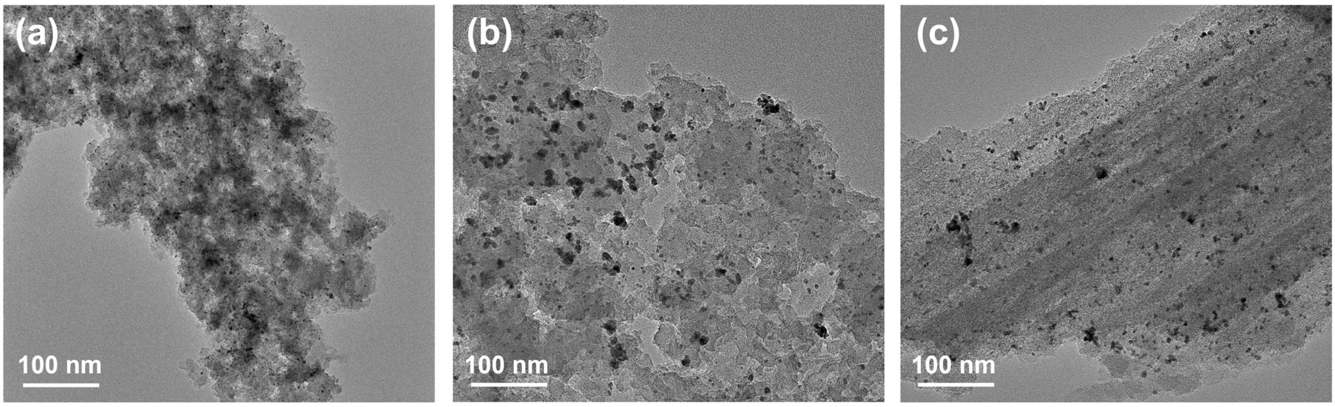

Figure S1: (a) TEM images of Pd@AC_0; (b) TEM images of PL-Pd@AC_{O2}; (c) TEM images of PL-Pd@AC_{Ar}

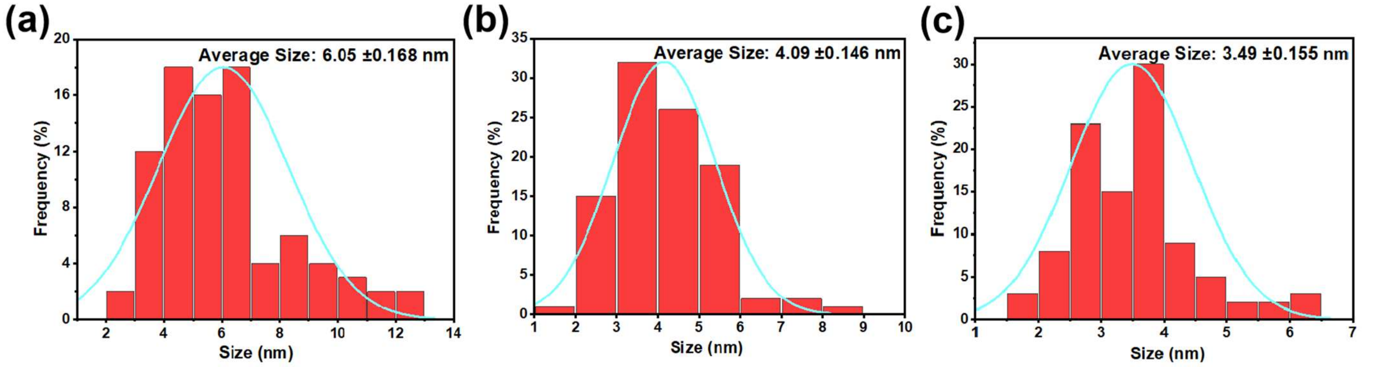

Figure S2: Particle size distribution histograms showing the average sizes of (a) Pd@AC$_0$, (b) PL-Pd@AC$_{O2}$, (c) PL-Pd@AC$_{Ar}$

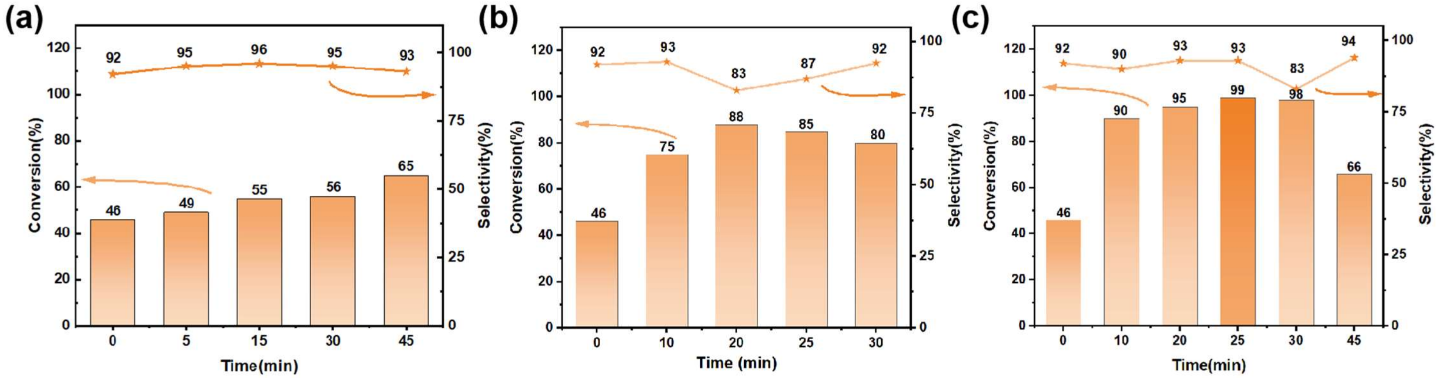

Figure S3: Effect of different plasma pretreatments on catalytic performance as a function of time: (a) Air plasma, (b) O_2 plasma, (c) Ar plasma.

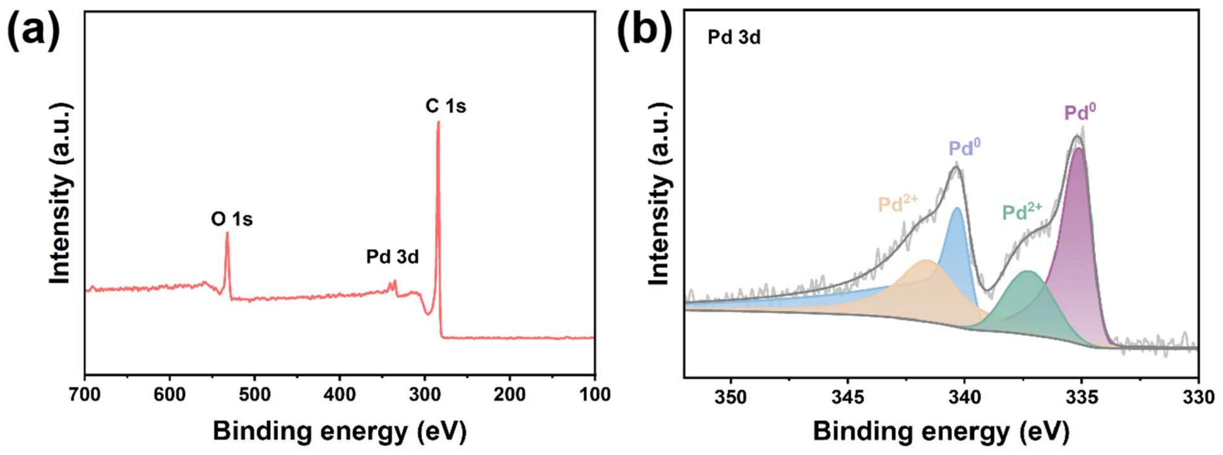

Figure S4: (a) XPS survey scan and (b) Pd 3d high-resolution spectrum of the recycled PL-Pd@AC$_{Ar}$ catalyst.

Table S1 Hydrogenation performance of PL-Pd@AC$_{Ar}$ for various hydroxyaromatic compounds

Entry	Substrate	Product	Time(h)	Conversion(%)	Selectivity(%)
1	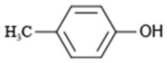	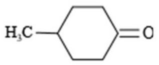	4	99.9	85.0
2	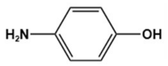	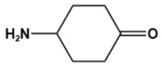	4	99.9	88.5
3	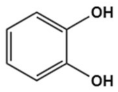	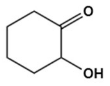	8	85.2	81.9
4	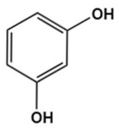	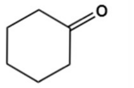	8	90.0	95.4
5	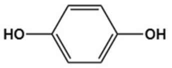	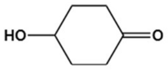	8	90.0	85.5
6	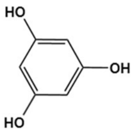	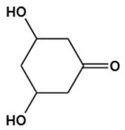	8	60.7	70.2
7	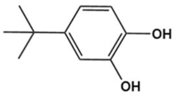	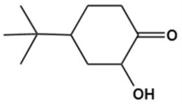	8	55.8	70.9

Reaction conditions: substrate (0.5 mmol), catalyst (20 mg), cyclohexane (10 mL), H_2 (1 MPa).
